# Large-Scale Brain Network Connectivity Mediates the Association Between Metabolic Risk Factors and Cognition: An fMRI Study of the Human Connectome Project

**DOI:** 10.5812/ijem-168867

**Published:** 2026-08-11

**Authors:** Yunus Soleymani, Seyed Amirhossein Batouli, Kazem Khalagi, Ata Pourabbasi

**Affiliations:** 1 Department of Neuroscience and Addiction Studies, School of Advanced Technologies in Medicine, Tehran University of Medical Sciences, Tehran, Iran; 2 Obesity and Eating Habits Research Center, Endocrinology and Metabolism Clinical Sciences Institute, Tehran University of Medical Sciences, Tehran, Iran; 3 Osteoporosis Research Center, Endocrinology and Metabolism Clinical Sciences Institute, Tehran University of Medical Sciences, Tehran, Iran; 4 Endocrinology and Metabolism Research Center, Endocrinology and Metabolism Clinical Sciences Institute, Tehran University of Medical Sciences, Tehran, Iran

**Keywords:** Metabolic Factors, Resting-state fMRI, Independent Component Analysis, Brain Networks, Cognition, Mediation Analysis

## Abstract

**Background:**

Subclinical metabolic disturbances have been associated with functional brain changes; however, the neural pathways mediating their cognitive effects remain unclear.

**Objectives:**

This study investigated whether large-scale brain network connectivity mediates associations between metabolic risk factors and cognition in healthy young adults.

**Methods:**

Data were obtained from 676 participants (22 - 37 years) in the Human Connectome Project. Metabolic indices (body mass index (BMI), glycated hemoglobin, thyroid-stimulating hormone (TSH), systolic/diastolic blood pressure, and hematocrit) were assessed alongside the National Institutes of Health (NIH) Toolbox Cognition Battery scores, the Mini-Mental State Examination, and fluid intelligence measures. Resting-state fMRI data underwent group independent component analysis to identify 14 intrinsic connectivity networks. Partial correlations were computed between network time series, and multiple regression models were adjusted for age, sex, race, and education. Mediation analyses with bootstrapped confidence intervals were conducted to test whether network connectivity explained the metabolic–cognition relationships.

**Results:**

We found that large-scale resting-state networks significantly mediated the associations between specific metabolic risk factors and cognitive performance. Specifically, BMI, hematocrit, and TSH showed significant associations with both cognition and network connectivity (all P < 0.05). BMI-related reductions in vocabulary performance were fully mediated by altered connectivity in the frontoparietal–language, sensorimotor–frontoparietal, primary visual–auditory, and default mode–executive attention networks and partially mediated (compensatory effect) by salience–sensorimotor connectivity (indirect effects: –0.0238, -0.0115, -0.0110, -0.0096, and 0.0068, respectively; 95% CIs: [–0.0421, –0.0085], [-0.0239, -0.0030], [-0.0237, -0.00016], [-0.0206, -0.0014], and [0.0001, 0.0165], respectively). Hematocrit’s positive association with vocabulary performance was partially mediated by the primary visual–auditory, salience–auditory, and language–dorsal attention networks, with primary visual–ventral attention connectivity exerting a suppressive effect (indirect effects: 0.0094, 0.0149, 0.0074, and -0.0113, respectively; 95% CIs: [0.0007, 0.0215], [0.0032, 0.0319], [0.0001, 0.0197], and [-0.0249, -0.0012], respectively). TSH was positively associated with fluid intelligence via a direct pathway (P = 0.010), without mediation by resting-state connectivity.

**Conclusions:**

These findings highlight large-scale brain networks as potential intermediate phenotypes that link metabolic health to cognition, suggesting that targeted neuromodulation of vulnerable circuits, combined with metabolic interventions, may offer novel strategies for preserving cognition in at-risk populations.

## 1. Background

Metabolic health is increasingly recognized as a pivotal determinant of brain integrity and cognitive resilience, even in the absence of overt clinical disease (1). Emerging evidence from large-scale population studies suggests that subclinical metabolic disturbances, such as elevated body mass index (BMI), hypertension, hematocrit imbalance, thyroid dysfunction, and impaired glycemic control, are associated not only with structural brain alterations (e.g., reduced gray matter volume, increased white matter hyperintensities, hippocampal atrophy) but also with functional disruptions in neural activity and connectivity patterns (2-4).

Functional magnetic resonance imaging (fMRI) studies have shown that these metabolic perturbations can reduce coherence among large-scale brain networks, including the default mode, salience, and frontoparietal control networks, which are essential for memory, attention, and executive function (5-7). These functional changes are not confined to older adults; rather, they may be detectable in early and mid-adulthood, underscoring the need to understand how metabolic risk factors shape brain function across the lifespan (8-11).

Resting-state fMRI (rs-fMRI) has enabled the identification of large-scale intrinsic connectivity networks that support domain-specific cognitive processes (6, 7). Independent component analysis (ICA), a data-driven approach, has proven particularly effective in delineating these networks without a priori assumptions, revealing reproducible patterns such as the default mode, frontoparietal control, and salience networks (12). Alterations in the functional integrity of these networks have been linked to metabolic dysfunction and cognitive impairment, suggesting that they may serve as intermediate phenotypes in the pathway from metabolic risk to cognitive decline (5, 7, 13).

Despite these advances, few studies have systematically examined whether intrinsic brain connectivity mediates the relationship between metabolic health and cognition (6, 7, 14). Addressing this gap, the present study employed a three-stage analytic framework to investigate the associations among metabolic risk factors, ICA-derived brain network connectivity, and cognitive performance in a large sample of young adults from the Human Connectome Project (HCP) (15). First, we assessed bivariate correlations between metabolic indices, including BMI, systolic and diastolic blood pressure (SBP and DBP), hematocrit, thyroid-stimulating hormone (TSH), and glycated hemoglobin (HbA1c), and both functional connectivity and performance across multiple cognitive domain scales. Second, we applied multiple regression models adjusted for age, sex, education, and race to identify robust associations. Finally, we conducted mediation analyses to determine which specific brain networks act as intermediaries linking metabolic health to cognitive outcomes.

## 2. Objectives

By integrating high-resolution neuroimaging, metabolic biomarkers, and domain-specific cognitive assessments, this study aims to advance our understanding of the neurofunctional pathways through which metabolic health influences cognition. The primary novelty of this research lies in its comprehensive, multisystem approach, which evaluates a broad panel of subclinical metabolic indices within a single, healthy cohort of young adults. Furthermore, rather than merely reporting parallel correlations, this study uniquely applies mediation analysis to identify large-scale resting-state brain networks as the bridge linking systemic metabolic health to cognitive performance. Ultimately, this approach underscores the potential of network-level biomarkers to inform early detection and guide precise intervention strategies (i.e., targeted neuromodulation approaches) aimed at preserving cognitive function in metabolically at-risk populations.

## 3. Methods

### 3.1. Participants

This study was conducted as a retrospective, cross-sectional secondary data analysis using the publicly available HCP-Young Adult (HCP-YA) dataset. All data were obtained from the HCP1200 Parcellation + Timeseries + Netmats (PTN) data release (16). Participants provided written informed consent under protocols approved by the Institutional Review Boards of Washington University in St. Louis and the University of Minnesota, in accordance with the Helsinki Declaration of 1975.

The cohort comprised healthy young adults aged 22 - 37 years, recruited from the general population and free of neuropsychiatric disorders and major medical illnesses such as cardiovascular disease and diabetes. The inclusion criteria for the current study were: 1) availability of the investigated metabolic risk factors; and 2) availability of processed resting-state fMRI data from the HCP1200 PTN release. The exclusion criteria were: 1) low-quality resting-state fMRI data; and 2) missing data for any specific metabolic marker, cognitive score, or covariate evaluated in the current study.

Because this study used a pre-existing, large-scale database, an a priori sample size calculation was not performed. Instead, the final sample size was determined by the maximum number of available participants who met the specified inclusion and exclusion criteria.

The data release included 1003 participants. Of these, 327 were excluded primarily because they had missing values for at least one dependent variable (cognitive scores) or independent variable (metabolic risk factors). We selected complete-case analysis rather than imputation to avoid assumptions regarding missingness mechanisms, acknowledging that this approach reduces sample size. Therefore, the final analytical sample comprised 676 participants with complete data.

All clinical, biological, and neuroimaging variables were originally assessed by trained personnel at the HCP consortium sites. All data collection, including MRI scanner calibration, biochemical assays of metabolic markers, and behavioral testing using standardized toolboxes, was conducted according to rigorously calibrated and standardized HCP protocols, the full details of which are published elsewhere (15, 16).

### 3.2. Metabolic Risk Factors

Metabolic risk factors included BMI, HbA1c, TSH, SBP, DBP, and hematocrit. All physiological data were collected by trained personnel and are available as Tier 2 Restricted variables within the HCP-Young Adults (HCP-YA) release.

BMI was calculated from measured height and weight using the formula:

$$\mathrm{BMI}=703\times \frac{\mathrm{weight}\mathrm{in}\mathrm{pounds}}{{\left(\mathrm{height}\mathrm{in}\mathrm{inches}\right)}^{2}}$$

HbA1c, expressed as a percentage of total hemoglobin, was derived from venous blood samples and served as an indicator of long-term glycemic control. TSH concentration, reported in milliunits per liter (mU/L), was measured using blood assays to assess thyroid function. Blood pressure was measured using standardized protocols, with SBP representing the maximum arterial pressure during cardiac contraction and DBP representing the minimum pressure during cardiac relaxation, both reported in millimeters of mercury (mmHg). Hematocrit, expressed as a percentage, represents the proportion of red blood cells in the total blood volume and was determined from blood samples. Two measurements were recorded for each participant, and the mean of these two measurements was used in the current study.

### 3.3. Cognition

The HCP protocol incorporates a comprehensive evaluation of cognitive abilities using the NIH Toolbox Cognition Battery (17). This assessment covers multiple cognitive domains, including language, executive function, episodic memory, processing speed, and working memory.

Seven specific tasks were used to measure these cognitive domains (18). Language skills were measured using the Picture Vocabulary (PicVocab) Test (vocabulary) and the Oral Reading Recognition (ReadEng) Test (reading decoding). Executive function was evaluated using the Dimensional Change Card Sort (CardSort) Test (cognitive flexibility) and the Flanker Inhibitory Control and Attention (Flanker) Test (inhibitory control and attention). Episodic memory was assessed using the Picture Sequence Memory (PicSeq) Test, processing speed using the Pattern Comparison Processing Speed (ProcSpeed) Test, and working memory using the List Sorting Working Memory (ListSort) Test. Because age was included as a covariate in subsequent statistical analyses, age-unadjusted scores were used in the present study.

In addition to the NIH Toolbox measures, two further cognitive assessments drawn from the HCP were included in this study (16). Global cognitive status was evaluated using the Mini-Mental State Examination (MMSE), a widely used screening tool for general cognitive functioning that assesses orientation, attention, memory, language, and visuospatial abilities. Although the MMSE is typically used as a screening tool in older adults, it was included here to provide a measure of global cognitive status for consistency across HCP assessments. Fluid intelligence (Gf) was measured using the Penn Progressive Matrices Test, which evaluates nonverbal reasoning and abstract problem-solving ability through pattern recognition and logical inference tasks. These measures provided complementary information to the NIH Toolbox scores, enabling a broader characterization of participants’ cognitive profiles.

### 3.4. Brain Connectivity

The procedures for data collection, preprocessing, and analysis for the PTN release in the Human Connectome Project have been described in detail elsewhere (16, 19, 20). The main processing steps are summarized below.

Participants completed four rs-fMRI runs, yielding a total of 58 minutes and 12 seconds of data per subject. Scans were acquired on a 3T Siemens Skyra using a multiband gradient-echo echo planar imaging (GE-EPI) sequence (factor = 8; TR = 720 ms; TE = 33.1 ms; flip angle = 52°; slice thickness = 2.0 mm). During acquisition, participants fixated on a crosshair presented on a dark background.

Data were drawn from the HCP 1200-PTN release and processed using the HCP minimal preprocessing pipeline; no additional preprocessing was applied. Dense connectomes were generated from individual time series using Incremental Group PCA in MELODIC (version 3.0, FMRIB, Oxford, UK) and were then parcellated using group ICA to derive 15 spatial network maps. Independent components (ICs) were labeled numerically with their associated anatomical regions. IC9 was excluded as noise, consistent with prior reports (21). Each IC was labeled according to the corresponding anatomical regions, guided by established neuroanatomical atlases (22-24). The major ICs and their associated large-scale brain networks are summarized in Table 1.

**Table 1.** Independent Components Identified in This Study and Their Corresponding Brain Networks and Cognitive Functions ^a^

IC#	Network Label	Cognitive Functional Role
**IC1**	Primary Visual Network	Early visual processing (V1, occipital cortex)
**IC2**	Default Mode Network	Self-referential thought, memory, internal mentation
**IC3**	Higher-Order Lateral Visual Network	Complex visual processing, object recognition
**IC4**	Sensorimotor Network (Leg/Trunk subdivision)	Motor control and somatosensory integration (lower limbs/trunk)
**IC5**	Frontoparietal Control Network (CEN/FPN)	Cognitive control, working memory, flexible adaptation
**IC6**	Salience Cingulo-Opercular Network	Detection of salient events, task-set maintenance
**IC7**	Language Network (Left-lateralized)	Language comprehension and production
**IC8**	Cerebellar Network	Motor coordination, sensorimotor prediction, learning
**IC9**	Noise (artifact component)	Likely physiological or scanner-related noise
**IC10**	Auditory Network	Auditory perception, speech and sound processing
**IC11**	Sensorimotor Network (Hand/Face subdivision)	Fine motor control and sensory processing (hand, face)
**IC12**	Medial Visual Network	Visual association, higher-order visual integration
**IC13**	Ventral Attention Network (Right-lateralized)	Stimulus-driven attention, reorienting responses
**IC14**	Dorsal Attention Network (Bilateral, secondary)	Goal-directed attention, visuospatial processing
**IC15**	Fronto-Insular Executive-Attention Network	Task control, switching, and salience integration

^a^ This table lists the brain networks and their corresponding cognitive roles; no statistical test was applied. Abbreviations: IC, independent component; CEN/FPN, central executive network/frontoparietal network.

Individual time series for each participant were aligned with the group-level ICA network maps. Dual regression was then applied by regressing the group spatial maps against the individual time series to generate subject-specific network matrices (netmats) that captured temporal fluctuations of each IC. Functional connectivity was then estimated between all pairs of IC networks. For this purpose, FSLNets (version 0.6, FMRIB, Oxford, UK) was used. Connectivity matrices were estimated as partial correlations between IC time series, with regularization applied via FSLNets to improve stability. The resulting functional connectivity matrices provided a subject-specific representation of coupling strength between resting-state networks.

### 3.5. Statistical Analysis

Given the exploratory nature of this study, our analytical strategy prioritized identifying potential associations and candidate pathways rather than confirmatory hypothesis testing (25). Accordingly, the findings should be considered hypothesis-generating and require replication in independent cohorts.

Metabolic indices (BMI, TSH, HbA1c, SBP, DBP, and hematocrit) were modeled as independent variables (X). Functional connectivity values between 14 major brain networks were defined as mediator variables (M), and cognitive performance (assessed using the NIH Toolbox Cognition Battery, the MMSE, and Fluid Intelligence) was treated as the dependent variable (Y). Age, sex, race, and education were included as covariates to account for potential confounding. Race was dummy-coded, and education was treated as a continuous variable (years of formal education). All variables were standardized to z-scores prior to analysis to harmonize measurement scales, enable direct comparison of coefficients, and enhance the numerical stability of the statistical models.

#### 3.5.1. Association Analysis

##### 3.5.1.1. Relationship Between Independent and Dependent Variables

At this stage, direct associations between metabolic indices (X) as independent variables and cognitive test scores (Y) as dependent variables were examined.

Pearson correlation coefficients were computed for each pair of variables (one metabolic factor and one cognitive measure), and correlation strength (r) and significance (P < 0.05) were reported. This step was intended to identify correlations between individual metabolic risk factors and cognitive performance.

Subsequently, only variables demonstrating significant correlations were entered into multiple linear regression models to assess the unique contribution of each metabolic factor to cognitive outcomes. In these models, the cognitive test score was specified as the dependent variable, the metabolic risk factor of interest as the predictor, and age, sex, race, and education were included as covariates to control for potential confounding. A P value < 0.05 was considered significant.

##### 3.5.1.2. Relationship Between Independent and Mediating Variables

At this stage, associations between metabolic indices (X) and functional connectivity measures (M) derived from rs-fMRI were examined.

Pearson correlation coefficients were calculated for each pair of variables (one metabolic factor and one connectivity measure), and statistical significance was assessed at P < 0.05. This analysis aimed to identify potential pathways through which metabolic risk factors might influence cognitive performance indirectly via alterations in brain connectivity patterns.

Subsequently, only variables showing significant correlations were entered into multiple linear regression models to assess the unique effects of each metabolic factor on connectivity strength. In these models, the functional connectivity measure served as the dependent variable, and the metabolic risk factor of interest was the independent variable. Age, sex, race, and education were included as covariates. Statistical significance was again set at P < 0.05.

##### 3.5.1.3. Significant Shared Relationships

At this stage, the results from the first two analyses were integrated to identify candidate mediation pathways. Specifically, only metabolic predictors (X) that showed significant direct associations with cognitive scores (Y) in the first stage, and significant associations with connectivity measures (M) in the second stage, were retained. This filtering procedure ensured that only meaningful X → M → Y pathways were carried forward into the mediation analysis, thereby reducing the likelihood of including spurious or non-significant effects.

#### 3.5.2. Mediation Analysis

Mediation analysis was performed using the standard three-variable model, in which X represented metabolic indices, M denoted functional connectivity measures, and Y indicated cognitive test scores. Covariates included age, sex, race, and education.

This model estimated three primary pathways (26): 1) the direct effect (Path c′), representing the influence of X on Y after controlling for M; 2) the indirect effect (Path a × b), representing the portion of X’s effect on Y transmitted through M; and 3) the total effect (Path c), representing the sum of direct and indirect effects. Moreover, path “a” refers to the metabolic risk factor–brain connectivity effect, and path “b” refers to the brain connectivity–cognitive score effect.

Indirect effects were estimated using a nonparametric bootstrapping procedure with 5000 resamples. Statistical significance of indirect effects was evaluated using 95% confidence intervals (CIs), with intervals excluding zero considered statistically significant. The significance of direct and total effects was determined using P values < 0.05. Because the primary objective of this study was exploratory, to identify candidate metabolic–connectivity–cognition pathways, we did not apply a correction for multiple comparisons at the initial screening stages (correlation and regression steps). Only associations that were subsequently confirmed in mediation analysis with bootstrapped CIs were considered significant.

**Figure 1.**
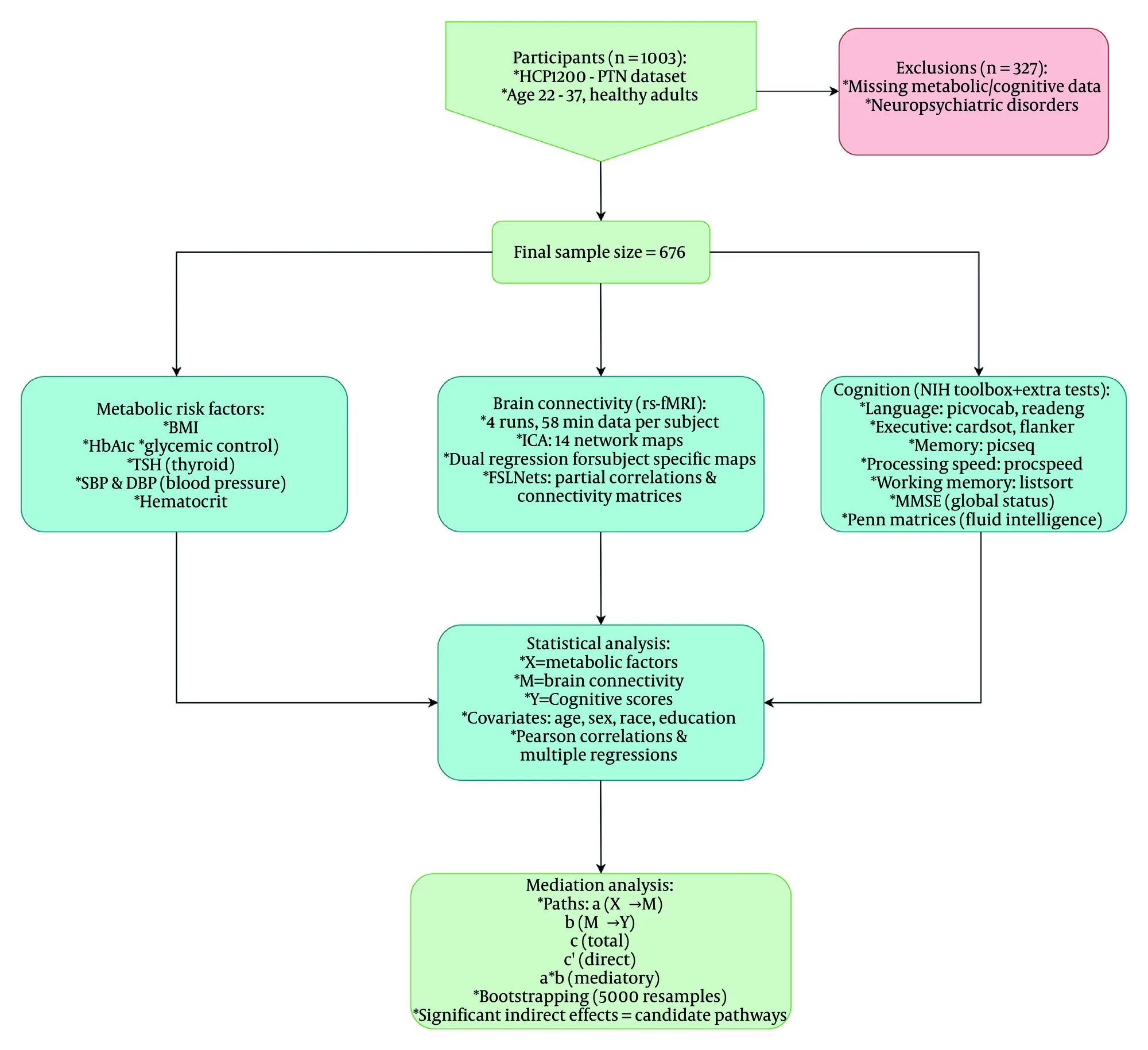
The flowchart of the current study. HCP 1200-PTN human connectome project 1200 parcellation+timeseries+netmats, BMI body mass index, HbA1C glycated hemoglobin, TSH thyroid-stimulating hormone, SBP systolic blood pressure, DBP diastolic blood pressure, NIH national institutes of health, PicVocab picture vocabulary, ReadEng oral reading recognition, CardSort dimensional change card sort, Flanker flanker inhibitory control and attention, PicSeq picture sequence memory, ProcSpeed pattern comparison processing speed, ListSort list sorting working memory, MMSE mini-mental state examination, Penn Matrices penn progressive matrices test, rs-fMRI resting state functional magnetic resonance imaging, ICA independent component analysis. Note: The diagram has been created using draw.io software (https://www.drawio.com).

All analyses were conducted using IBM SPSS Statistics (version 27) and the PROCESS macro (version 4.2; Andrew F. Hayes), which enables the simultaneous estimation of direct, indirect, and total effects, along with bootstrapped confidence intervals. All mediation models were tested for assumptions of linearity, independence, and multicollinearity. No violations were observed. Figure 1 presents the flowchart of the multiple steps conducted in the current study.

## 4. Results

### 4.1. Participant Characteristics

The final sample comprised 676 participants (48.7% female; mean age = 28.66 ± 3.65 years; mean years of education = 15.03 ± 1.71). Most participants were white (77.4%). Table 2 presents the demographic, cognitive, and metabolic characteristics of the cohort.

**Table 2.** Demographic and Clinical Characteristics of the Study Participants ^a, b^

Variables	Values
**Sample Size**	676
**Age (y)**	28.66 ± 3.65
**Sex (Female)**	329 (48.7)
**Education (y)**	15.03 ± 1.71
**Race**	
White	523 (77.4)
Others	153 (22.6)
**Metabolic**	
BMI	25.99 ± 4.53
HbA1c (%)	5.23 ± 0.33
TSH (mU/L)	1.78 ± 1.13
Systolic BP (mmHg)	123.47 ± 13.55
Diastolic BP (mmHg)	76.33 ± 10.59
Hematocrit (%)	44.18 ± 4.50
**Cognition Tests**	
MMSE	29.06 ± 0.99
Penn Progressive Matrices (Gf)	17.17 ± 4.62
**NIH Toolbox Cognition Battery**	
PicVocab	117.58 ± 9.24
ReadEng	117.74 ± 10.39
CardSort	115.96 ± 10.26
Flanker	111.92 ± 10.23
PicSeq	112.39 ± 13.12
ProcSpeed	115.67 ± 15.76
ListSort	111.68 ± 11.13

^a^ Values are expressed as No., mean ± SD or No. (%). Abbreviations: SD, standard deviation; BMI, body mass index; HbA1c, glycated hemoglobin; TSH, thyroid-stimulating hormone; mU/L, milliunits per liter; BP, blood pressure; mmHg, millimeters of mercury; MMSE, Mini-Mental State Examination; Gf, fluid intelligence; NIH, National Institutes of Health; PicVocab, Picture Vocabulary; ReadEng, Oral Reading Recognition; CardSort, Dimensional Change Card Sort; Flanker, Flanker Inhibitory Control and Attention; PicSeq, Picture Sequence Memory; ProcSpeed, Pattern Comparison Processing Speed; ListSort, List Sorting Working Memory.

^b^ Descriptive statistics were used to summarize demographic and clinical characteristics.

### 4.2. Preliminary Analysis Findings

As described in the Materials and Methods (section 3.5.1.3), only metabolic risk factors showing significant associations with both brain connectivity and cognitive performance scores are presented here. These findings formed the basis for the subsequent mediation analysis. Preliminary analyses identified BMI, hematocrit, and TSH as metabolic risk factors significantly associated with both cognitive performance and brain connectivity. These associations are detailed in Table S1 in the Supplementary File.

#### 4.2.1. Relationship Between Cognitive Scores and Metabolic Risk Factors

Higher BMI (β = -0.069, P = 0.046) and higher hematocrit (β = 0.115, P = 0.004) were significantly associated with lower and higher PicVocab scores, respectively. Higher TSH levels (β = 0.095, P = 0.010) were significantly associated with higher Gf scores.

#### 4.2.2. Relationship Between Brain Connectivity and BMI

In analyses of brain connectivity, BMI was significantly associated with the network connectivity of IC1,6 (primary visual-salience, β = -0.121, P = 0.001); IC1,10 (primary visual–auditory, β = 0.133, P < 0.001); IC1,11 (primary visual-sensorimotor, β = 0.078, P = 0.048); IC1,14 (primary visual-dorsal attention, β = -0.078, P = 0.046); IC2,3 (default mode network (DMN)-lateral visual, β = -0.103, P = 0.008); IC2,7 (DMN-language, β = 0.076, P = 0.045); IC2,11 (DMN-sensorimotor, β = 0.122, P = 0.001); IC2,15 (DMN-executive attention, β = 0.107, P = 0.006); IC3,4 (lateral visual-sensorimotor, β = -0.081, P = 0.040); IC3,8 (lateral visual-cerebellar, β = -0.081, P = 0.034); IC3,10 (lateral visual-auditory, β = 0.156, P < 0.001); IC4,5 (sensorimotor–frontoparietal, β = 0.117, P = 0.002); IC5,7 (frontoparietal-language, β = 0.129, P = 0.001); IC5,8 (frontoparietal-cerebellar, β = -0.084, P = 0.030); IC5,10 (frontoparietal-auditory, β = 0.124, P = 0.001); IC6,11 (salience-sensorimotor, β = 0.101, P = 0.010); IC6,14 (salience-dorsal attention, β = 0.144, P < 0.001); IC7,13 (language-ventral attention, β = 0.123, P = 0.001); IC7,15 (language–executive attention, β = 0.119, P = 0.002); IC8,14 (cerebellar-dorsal attention, β = -0.086, P = 0.028); IC10,13 (auditory-ventral attention, β = 0.156, P < 0.001); and IC12,14 (medial visual–dorsal attention, β = 0.078, P = 0.047).

#### 4.2.3. Relationship Between Brain Connectivity and Hematocrit

Hematocrit was significantly associated with the network connectivity of IC1,10 (primary visual-auditory, β = -0.116, P = 0.010); IC1,13 (primary visual-ventral attention, β = -0.099, P = 0.027); IC2,10 (DMN-auditory, β = -0.106, P = 0.018); IC5,11 (frontoparietal-sensorimotor, β = -0.088, P = 0.049); IC6,10 (salience-auditory, β = -0.119, P = 0.008); IC6,11 (salience-sensorimotor, β = 0.126, P = 0.005); IC7,14 (language-dorsal attention, β = 0.096, P = 0.032); and IC12,13 (medial visual-ventral attention, β = 0.088, P = 0.049).

#### 4.2.4. Relationship Between Brain Connectivity and TSH

TSH was significantly associated with the network connectivity of IC1,2 (primary visual-DMN, β = 0.082, P = 0.032); IC3,5 (lateral visual-frontoparietal, β = -0.096, P = 0.011); and IC5,10 (frontoparietal-auditory, β = 0.086, P = 0.026).

### 4.3. Mediation Analysis Findings

The significant mediating pathways identified in this study are summarized below. Detailed mediation analysis results are presented in Table 3.

**Table 3.** Mediation Analysis Results for Metabolic–Connectivity–Cognition Pathways ^a^

Predictors (X) and Mediators (M)	Outcome (Y)	Direct Effect (c′)	Indirect (mediatory) Effect (a × b, 95% CI)	Total Effect (c)
**BMI**	PicVocab			- 0.0698
CE & Language connectivity		– 0.0460	– 0.0238 (– 0.0421, –0.0085)	
CE & Cerebellar connectivity		- 0.0654	- 0.0044 (- 0.0135, 0.0014)	
CE & Auditory connectivity		- 0.0732	0.0033 (-0.0055, 0.0129)	
Salience & Sensorimotor connectivity		- 0.0766	0.0068 (0.0001, 0.0165)	
Salience & DA connectivity		- 0.0754	0.0056 (-0.0041, 0.0169)	
Language & VA connectivity		- 0.0699	0.0000 (-0.0009, 0.0086)	
Language & EA connectivity		- 0.0661	- 0.0037 (-0.0138, 0.0040)	
PV & Salience connectivity		- 0.0643	- 0.0056 (- 0.0159, 0.0022)	
DMN & Lateral visual connectivity		- 0.0671	- 0.0027 (- 0.0123, 0.0046)	
DMN & Language connectivity		- 0.0680	- 0.0018 (- 0.0084, 0.0036)	
LV & Sensorimotor connectivity		- 0.0711	0.0013 (- 0.0044, 0.0079)	
LV & Cerebellar connectivity		- 0.0681	- 0.0017 (- 0.0088, 0.0044)	
Sensorimotor & CE connectivity		- 0.0583	- 0.0115 (- 0.0239, - 0.0030)	
PV & Auditory connectivity		- 0.0589	- 0.0110 (- 0.0237, -0.0016)	
PV & Sensorimotor connectivity		- 0.0699	0.0001 (- 0.0061, 0.0060)	
PV & DA connectivity		- 0.0650	- 0.0048 (- 0.0146, 0.0007)	
DMN & Sensorimotor connectivity		- 0.0682	- 0.0016 (- 0.0113, 0.0076)	
DMN & EA connectivity		- 0.0602	- 0.0096 (- 0.0206, - 0.0014)	
LV & Auditory connectivity		- 0.0612	- 0.0087 (- 0.0215, 0.0020)	
Cerebellar & DA connectivity		- 0.0730	0.0031 (- 0.0026, 0.0117)	
**Hematocrit**	PicVocab			0.1159
PV & Auditory connectivity		0.1066	0.0094 (0.0007, 0.0215)	
PV & VA connectivity		0.1272	- 0.0113 (- 0.0249, - 0.0012)	
DMN & Auditory connectivity		0.1139	0.0021 (- 0.0064, 0.0109)	
CE & Sensorimotor connectivity		0.1185	- 0.0026 (- 0.0111, 0.0040)	
Salience & Auditory connectivity		0.1011	0.0149 (0.0032, 0.0319)	
Salience & Sensorimotor connectivity		0.1096	0.0063 (- 0.0017, 0.0189)	
Language & DA connectivity		0.1085	0.0074 (0.0001, 0.0197)	
MV & VA connectivity		0.1163	- 0.0003 (- 0.0072, 0.0197)	
**TSH**	Gf			0.0951
PV & DMN connectivity		0.0991	- 0.0041 (- 0.0160, 0.0023)	
LV & CE connectivity		0.0940	0.0011 (-0.0070, 0.0103)	
CE & Auditory connectivity		0.0957	- 0.0007 (- 0.0082, 0.0065)	

^a^ The direct effect (Path c′) represents the influence of X (predictor) on Y (outcome) after controlling for M (mediator). The indirect effect (Path a × b) represents the portion of X’s effect on Y transmitted through M. The total effect (Path c) is the sum of the direct and indirect effects. Standardized coefficients are reported. Mediation analysis using the PROCESS macro was performed to calculate direct and indirect effects, adjusting for age, sex, race, and education. P < 0.05 was considered significant for direct effects. Statistical significance of indirect effects was tested using bootstrapped confidence intervals (CIs) with 5000 resamples; CIs excluding zero were considered significant. Abbreviations: CI, confidence interval; BMI, body mass index; TSH, thyroid-stimulating hormone; CE, central executive; DA, dorsal attention; VA, ventral attention; EA, executive attention; PV, primary visual; DMN, default mode network; LV, lateral visual; MV, medial visual; PicVocab, Picture Vocabulary test performance; Gf, fluid intelligence test performance.

#### 4.3.1. BMI–Connectivity–Cognition Pathways

**Figure 2.**
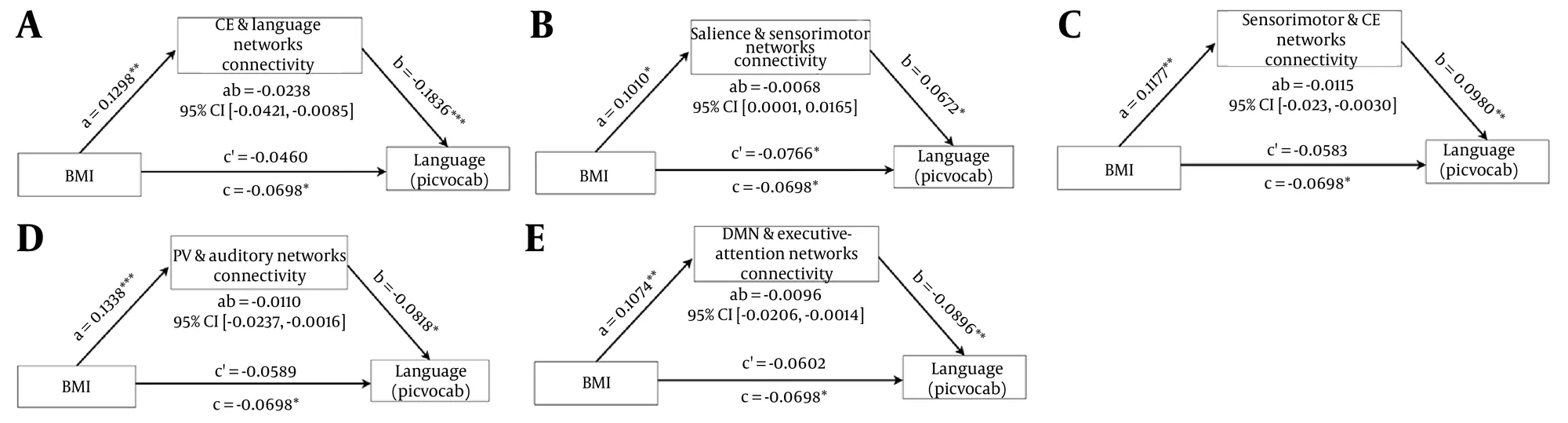
Significant mediation findings in relation to the BMI-Connectivity-Cognition pathway. BMI body mass index, CE central executive, CI confidence interval, PicVocab picture vocabulary, PV primary visual, DMN default mode network. Note: The figure has been created using draw.io software (https://www.drawio.com).

Mediation analysis showed that the association between BMI and PicVocab performance was significantly mediated by frontoparietal (central executive)–language network connectivity (indirect effect = –0.0238, 95% CI [–0.0421, –0.0085], accounting for 34% of the total effect size). No significant direct effect remained after accounting for the mediator (c′ = –0.0460, P > 0.05), suggesting that the relationship was fully mediated by connectivity (Figure 2A).

The association between BMI and PicVocab performance was significantly mediated by salience–sensorimotor network connectivity (indirect effect = 0.0068, 95% CI [0.0001, 0.0165], accounting for about 10% of the total effect size). The direct effect remained significant after accounting for the mediator (c′ = –0.0766, P < 0.05), suggesting that the relationship was partially mediated by connectivity. Notably, although higher BMI was directly associated with lower PicVocab performance, it also exerted a small positive indirect effect through salience–sensorimotor connectivity, which slightly attenuated the negative direct association, yielding an overall compensatory-type mediation (Figure 2B).

The association between BMI and PicVocab performance was significantly mediated by sensorimotor-frontoparietal (central executive) network connectivity (indirect effect = –0.0115, 95% CI [–0.0239, –0.0030], accounting for 16% of the total effect size). No significant direct effect remained after accounting for the mediator (c′ = –0.0583, P > 0.05), suggesting that the relationship was fully mediated by connectivity (Figure 2C).

The association between BMI and PicVocab performance was significantly mediated by primary visual-auditory network connectivity (indirect effect = –0.0110, 95% CI [–0.0237, –0.0016], accounting for about 16% of the total effect size). No significant direct effect remained after accounting for the mediator (c′ = –0.0589, P > 0.05), suggesting that the relationship was fully mediated by connectivity (Figure 2D).

The association between BMI and PicVocab performance was significantly mediated by DMN-executive attention network connectivity (indirect effect = –0.0096, 95% CI [–0.0206, –0.0014], accounting for about 14% of the total effect size). No significant direct effect remained after accounting for the mediator (c′ = –0.0602, P > 0.05), suggesting that the relationship was fully mediated by connectivity (Figure 2E).

#### 4.3.2. Hematocrit–Connectivity–Cognition Pathways

**Figure 3.**
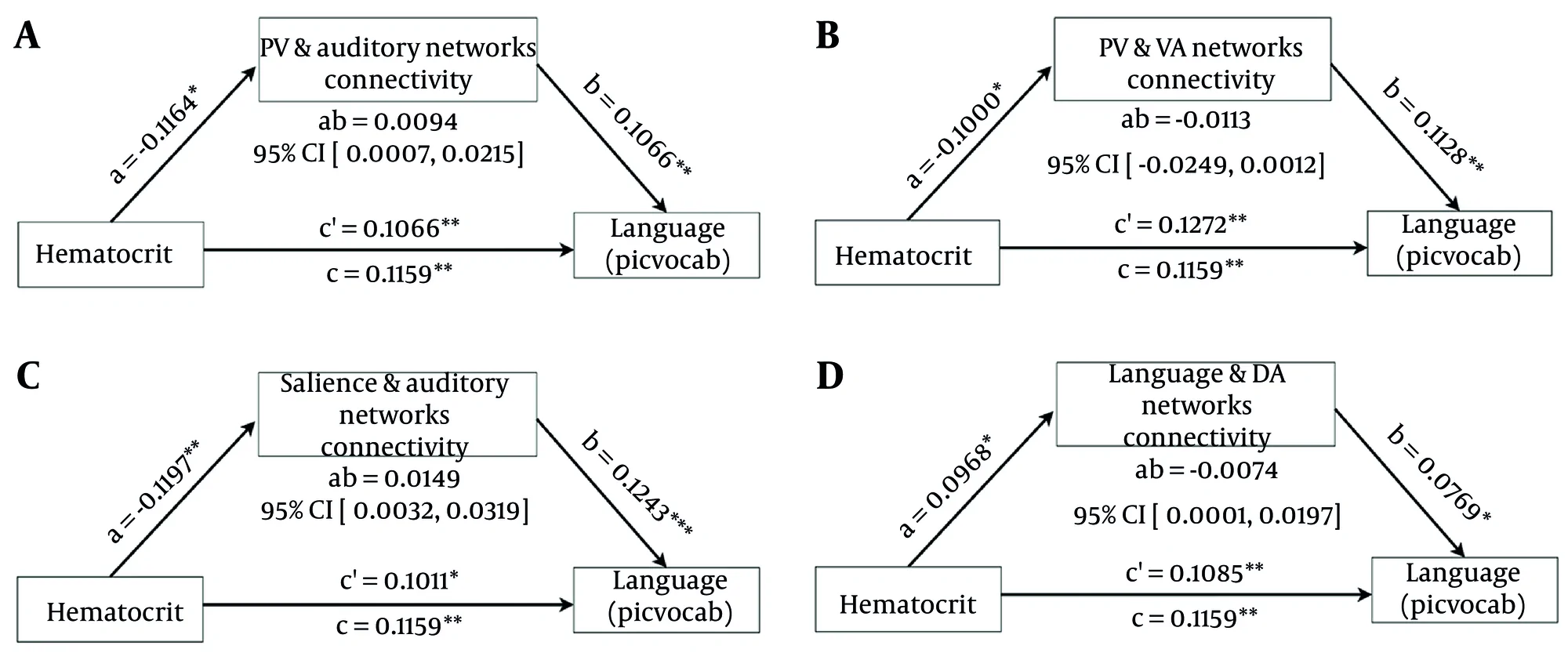
Significant mediation findings in relation to the Hematocrit- Connectivity-Cognition pathway. PV primary visual, CI confidence interval, PicVocab picture vocabulary, VA ventral attention, DA dorsal attention. Note: The figure has been created using draw.io software (https://www.drawio.com).

The association between hematocrit and PicVocab performance was significantly mediated by primary visual–auditory network connectivity (indirect effect = 0.0094, 95% CI [0.0007, 0.0215], accounting for 8% of the total effect size). The direct effect remained significant after accounting for the mediator (c′ = 0.1066, P < 0.01), suggesting that the relationship was partially mediated by connectivity (Figure 3A).

The association between hematocrit and PicVocab performance was significantly mediated by primary visual–ventral attention network connectivity (indirect effect = -0.0113, 95% CI [-0.0249, -0.0012], accounting for about 10% of the total effect size). The direct effect remained significant after accounting for the mediator (c′ = 0.1272, P < 0.01), suggesting that the relationship was partially mediated by connectivity. Notably, hematocrit showed a positive direct association with PicVocab performance, whereas a negative indirect pathway via primary visual–ventral attention connectivity partially suppressed this effect, yielding an overall suppression-type mediation (Figure 3B).

The association between hematocrit and PicVocab performance was significantly mediated by salience–auditory network connectivity (indirect effect = 0.0149, 95% CI [0.0032, 0.0319], accounting for about 13% of the total effect size). The direct effect remained significant after accounting for the mediator (c′ = 0.1011, P < 0.05), suggesting that the relationship was partially mediated by connectivity (Figure 3C).

The association between hematocrit and PicVocab performance was significantly mediated by language–dorsal attention network connectivity (indirect effect = 0.0074, 95% CI [0.0001, 0.0197], accounting for 6% of the total effect size). The direct effect remained significant after accounting for the mediator (c′ = 0.1085, P < 0.01), suggesting that the relationship was partially mediated by connectivity (Figure 3D).

#### 4.3.3. TSH–Connectivity–Cognition Pathways

For TSH, no significant indirect effect via network connectivity was observed, indicating that the association with fluid intelligence ability was not mediated by connectivity. This pattern suggests a primarily direct relationship between TSH and fluid intelligence performance (Table 3).

## 6. Discussion

The present study provides novel insights into the brain network-mediated pathways through which metabolic risk factors influence cognitive performance in young adults. By integrating high-resolution rs-fMRI with comprehensive metabolic and cognitive measures from the HCP, we identified distinct connectivity-based mediating mechanisms for BMI and hematocrit, while also uncovering a primarily direct effect of TSH on cognition. Collectively, these findings highlight the complexity of metabolic–cognitive associations and underscore the critical role of large-scale brain networks as intermediate phenotypes in this relationship. Importantly, the identified mediating brain network connectivity patterns offer promising targets for neuromodulation interventions aimed at mitigating the detrimental impact of metabolic risk factors on cognitive performance.

Resting-state fMRI studies have repeatedly shown that obesity is associated with altered functional connectivity within and between core brain networks that support high-level cognitive functions (27). Higher BMI correlates with selective changes in functional connectivity involving attentional and default-mode networks, suggesting disrupted synchrony in circuits that subserve both internally and externally directed cognition (28, 29). In particular, individuals with obesity often exhibit greater intrinsic connectivity within the salience network, especially involving paralimbic and striatal regions, coupled with diminished connectivity between the salience network and both the DMN and executive control networks, including frontoparietal systems (30-32).

Consistent with previous studies (30, 32), our results revealed a link between higher BMI and lower language-related cognitive performance, particularly evident in vocabulary tasks. Mediation analysis indicated that this relationship was explained by altered connectivity between multiple large-scale networks, including frontoparietal–language, sensorimotor–frontoparietal, primary visual–auditory, and DMN–executive attention networks. The full mediation observed for some of these pathways suggests that the BMI–cognition association reflects the cognitive impact of excess adiposity operating predominantly through disrupted network integration, rather than through direct metabolic–cognitive effects. Interestingly, the salience–sensorimotor connectivity pathway exhibited a compensatory role, partially mitigating the detrimental impact of BMI on vocabulary. This pattern may reflect the adaptive recruitment of alternative circuits to maintain performance when primary language networks are strained, in line with previous structural volumetry studies (33). These pathway-specific findings extend prior studies that primarily focused on the Default Mode and Executive networks (28-32), suggesting that obesity-related cognitive changes may also depend on the functional reorganization of primary sensory and motor networks. However, more evidence is needed to support this claim.

Hematocrit levels demonstrated a positive association with vocabulary performance, but several connectivity pathways partially mediated this relationship. In particular, connectivity between the primary visual and ventral attention networks exerted a suppressive effect, whereas connectivity between the salience and auditory networks and between the language and dorsal attention networks contributed positively. This pattern suggests that hematocrit influences cognition through both facilitative and inhibitory neural mechanisms, consistent with its complex role in regulating cerebral oxygenation and blood viscosity (34). This suppression-type mediation is particularly noteworthy, as it indicates that elevated hematocrit can simultaneously support cognitive performance directly while being counterbalanced by maladaptive network reorganization (35). Accordingly, our results align with earlier physiological studies demonstrating that, although higher hematocrit increases oxygen-carrying capacity, it also increases blood viscosity, which can selectively alter cerebral blood flow in highly metabolically active hubs such as the visual and attentional networks (5, 10, 34-36). These nuanced findings underscore the importance of considering brain network dynamics when evaluating hematological contributions to cognition.

Unlike BMI and hematocrit, we found that TSH was associated with fluid intelligence through a direct relationship, and we did not identify brain network connectivity as a mediator in this pathway. This finding suggests that thyroid function may influence cognition through mechanisms not captured by large-scale resting-state networks, such as cellular metabolism, synaptic plasticity, or neurovascular coupling (37-39). Previous studies suggest that thyroid dysfunction can affect brain structure and function, but these effects may emerge only during cognitive tasks or in specific brain regions (37, 40, 41). While prior fMRI research has frequently demonstrated widespread resting-state connectivity disruptions in patients with overt clinical hypothyroidism (38, 40), our study focused on individuals with TSH variations primarily within the normal physiological range. Consequently, in the absence of severe pathology, these subtle TSH fluctuations might primarily modulate local molecular efficiency rather than inducing the macroscopic network reorganization observed in clinical populations. These distinctions highlight the importance of differentiating between subclinical metabolic variations and overt disease states when interpreting neurocognitive pathways. This also suggests that subtler or task-specific network alterations may mediate TSH–cognition relationships but are not detectable in large-scale resting-state frameworks.

Collectively, these findings provide compelling support for the notion that metabolic risk factors affect cognition through heterogeneous pathways, some reliant on large-scale network integrity and others independent of it. The results also emphasize the importance of early and subclinical metabolic changes, as the studied cohort comprised healthy young adults without overt disease. This raises concerns that even mild deviations in BMI, hematocrit, or thyroid function can leave detectable imprints on neural connectivity and cognition decades before clinical manifestations emerge.

A particularly promising approach for future research is the application of neuromodulatory techniques to mitigate the cognitive consequences of metabolic dysfunction. Non-invasive brain stimulation methods, such as transcranial magnetic stimulation (TMS), transcranial direct current stimulation (tDCS), and more advanced approaches such as transcranial alternating current stimulation (tACS) and focused ultrasound neuromodulation, have demonstrated the capacity to enhance functional connectivity and improve domain-specific cognitive functions (42, 43). Given our findings that metabolic (specifically BMI)-related cognitive impairments are mediated by disruptions in networks such as the frontoparietal control, sensorimotor, salience, and language systems, targeted neuromodulation of these circuits may represent a novel strategy for preserving or restoring cognitive function in metabolically at-risk populations. Furthermore, combining neuromodulation with lifestyle interventions (e.g., diet, exercise) could yield synergistic effects by addressing both systemic metabolic dysfunction and its neural consequences.

The present study has several notable strengths, including the integration of high-resolution resting-state fMRI with comprehensive metabolic and cognitive measures from a well-characterized cohort. This approach enabled the evaluation of early, subclinical neural alterations in healthy young adults. The application of mediation analysis provided a nuanced understanding of the complex direct and indirect, network-mediated pathways linking metabolic risk factors to cognition. However, several limitations must be acknowledged. First, the cross-sectional design precludes causal inference; longitudinal studies are needed to confirm whether metabolic changes lead to progressive connectivity alterations and cognitive decline. Second, the focus on resting-state connectivity may not capture task-specific dynamics that are equally relevant to cognition. Third, while our primary goal was to identify candidate metabolic–connectivity–cognition pathways rather than to make definitive inferences, replication in independent samples with correction for multiple comparisons is necessary. Finally, we studied young adults, and the findings may not generalize to older populations in whom metabolic and cognitive changes are more pronounced.

### 5.1. Conclusions

In conclusion, this study demonstrates that BMI and hematocrit influence cognition primarily through alterations in large-scale brain network connectivity. In contrast, no such mediation effects were found in relation to TSH. These findings highlight the central role of network-mediated mechanisms in metabolic–cognitive interactions and suggest novel opportunities for intervention. Future research should explore whether targeted neuromodulation of vulnerable networks can offset the detrimental cognitive effects of metabolic risk factors, potentially paving the way for precision medicine approaches to brain health.

ijem-24-4-168867-s001.pdf

## Data Availability

Data of this study were provided by the Human Connectome Project, WU-Minn Consortium (Principal Investigators: David Van Essen and Kamil Ugurbil; 1U54MH091657) and was funded by the 16 NIH Institutes and Centers that support the NIH Blueprint for Neuroscience Research; and by the McDonnell Center for Systems Neuroscience at Washington University. The data are publicly available at https://www.humanconnectome.org/study/hcp-young-adult/data-releases.

## References

[1] Schwartz SS, Herman ME, Tun MTH, Barone E, Butterfield DA (2024). The double life of glucose metabolism: brain health, glycemic homeostasis, and your patients with type 2 diabetes. BMC Medicine.

[2] Qureshi D, Topiwala A, Al Abid SU, Allen NE, Kuźma E, Littlejohns TJ (2024). Association of Metabolic Syndrome With Neuroimaging and Cognitive Outcomes in the UK Biobank. Diabetes Care.

[3] Shen C, Liu C, Qiu A (2023). Metabolism-related brain morphology accelerates aging and predicts neurodegenerative diseases and stroke: a UK Biobank study. Transl Psychiatry.

[4] Zhao S, Wu J, Liu X, Du Y, Wang X, Xia Y (2025). Exploring the interaction effects of subclinical hypothyroidism and major depressive disorder on brain networks. BMC Medicine.

[5] Edwin Thanarajah S (2025). Midlife Dietary Quality and Body Composition Relevance for Brain Connectivity and Cognitive Performance in Later Life. JAMA Network Open.

[6] Ranasinghe P, Mapa MST (2024). Functional connectivity and cognitive decline: a review of rs-fMRI, EEG, MEG, and graph theory approaches in aging and dementia. Exploration of Medicine.

[7] Voigt K, Liang EX, Misic B, Ward PGD, Egan GF, Jamadar SD (2023). Metabolic and functional connectivity provide unique and complementary insights into cognition-connectome relationships. Cereb Cortex.

[8] Rashid B, Glasser MF, Nichols T, Van Essen D, Juttukonda MR, Schwab NA (2023). Cardiovascular and metabolic health is associated with functional brain connectivity in middle-aged and older adults: Results from the Human Connectome Project-Aging study. NeuroImage.

[9] Haase Alasantro L, Hicks TH, Green-Krogmann E, Murphy C (2021). Metabolic syndrome and cognitive performance across the adult lifespan. PLOS ONE.

[10] García-García I, Donica O, Cohen AA, Gonseth Nusslé S, Heini A, Nusslé S (2023). Maintaining brain health across the lifespan. Neuroscience & Biobehavioral Reviews.

[11] Bangen KJ, Armstrong NM, Au R, Gross AL (2019). Metabolic Syndrome and Cognitive Trajectories in the Framingham Offspring Study. Journal of Alzheimer's Disease.

[12] Jung J, Lambon Ralph MA (2023). Distinct but cooperating brain networks supporting semantic cognition. Cereb Cortex.

[13] Yang SQ, Xu ZP, Xiong Y, Zhan YF, Guo LY, Zhang S (2016). Altered Intranetwork and Internetwork Functional Connectivity in Type 2 Diabetes Mellitus With and Without Cognitive Impairment. Sci Rep.

[14] Soleymani Y, Batouli SAH, Ahangar AA, Pourabbasi A (2024). Association of glycosylated hemoglobin concentrations with structural and functional brain changes in the normoglycemic population: A systematic review. J Neuroendocrinol.

[15] Elam JS, Glasser MF, Harms MP, Sotiropoulos SN, Andersson JLR, Burgess GC (2021). The Human Connectome Project: A retrospective. Neuroimage.

[16] Van Essen DC, Ugurbil K, Auerbach E, Barch D, Behrens TE, Bucholz R (2012). The Human Connectome Project: a data acquisition perspective. Neuroimage.

[17] Gershon RC, Wagster MV, Hendrie HC, Fox NA, Cook KF, Nowinski CJ (2013). NIH toolbox for assessment of neurological and behavioral function. Neurology.

[18] Heaton RK, Akshoomoff N, Tulsky D, Mungas D, Weintraub S, Dikmen S (2014). Reliability and validity of composite scores from the NIH Toolbox Cognition Battery in adults. J Int Neuropsychol Soc.

[19] Sadler JR, Shearrer GE, Burger KS (2018). Body mass variability is represented by distinct functional connectivity patterns. Neuroimage.

[20] Glasser MF, Sotiropoulos SN, Wilson JA, Coalson TS, Fischl B, Andersson JL (2013). The minimal preprocessing pipelines for the Human Connectome Project. Neuroimage.

[21] Sadler JR, Shearrer GE, Burger KS (2021). Alterations in ventral attention network connectivity in individuals with prediabetes. Nutr Neurosci.

[22] Smith SM, Fox PT, Miller KL, Glahn DC, Fox PM, Mackay CE (2009). Correspondence of the brain's functional architecture during activation and rest. Proc Natl Acad Sci U S A.

[23] Yeo BT, Krienen FM, Sepulcre J, Sabuncu MR, Lashkari D, Hollinshead M (2011). The organization of the human cerebral cortex estimated by intrinsic functional connectivity. J Neurophysiol.

[24] Shirer WR, Ryali S, Rykhlevskaia E, Menon V, Greicius MD (2012). Decoding subject-driven cognitive states with whole-brain connectivity patterns. Cereb Cortex.

[25] Ranstam J (2019). Hypothesis-generating and confirmatory studies, Bonferroni correction, and pre-specification of trial endpoints. Acta Orthop.

[26] Hu Y, Ji G, Li G, Manza P, Zhang W, Wang J (2021). Brain Connectivity, and Hormonal and Behavioral Correlates of Sustained Weight Loss in Obese Patients after Laparoscopic Sleeve Gastrectomy. Cereb Cortex.

[27] Syan SK, McIntyre-Wood C, Minuzzi L, Hall G, McCabe RE, MacKillop J (2021). Dysregulated resting state functional connectivity and obesity: A systematic review. Neuroscience & Biobehavioral Reviews.

[28] Parsons N, Steward T, Clohesy R, Almgren H, Duehlmeyer L (2022). A systematic review of resting-state functional connectivity in obesity: Refining current neurobiological frameworks and methodological considerations moving forward. Reviews in Endocrine and Metabolic Disorders.

[29] Coveleskie K, Gupta A, Kilpatrick LA, Mayer ED, Ashe-McNalley C, Stains J (2015). Altered functional connectivity within the central reward network in overweight and obese women. Nutrition & Diabetes.

[30] Ottino-González J, Baggio HC, Jurado M, Segura B, Caldú X, Prats-Soteras X (2021). Alterations in Brain Network Organization in Adults With Obesity as Compared With Healthy-Weight Individuals and Seniors. Biopsychosocial Science and Medicine.

[31] Legget KT, Wylie KP, Berman BD, Tregellas JR (2021). Altered between-network connectivity in individuals prone to obesity. Physiology & Behavior.

[32] Byeon K, Lee MJ, Morys F (2020). Whole-brain functional connectivity correlates of obesity phenotypes. Human Brain Mapping.

[33] Li L, Yu H, Zhong M, Liu S, Wei W, Meng Y (2022). Gray matter volume alterations in subjects with overweight and obesity: Evidence from a voxel-based meta-analysis. Frontiers in Psychiatry.

[34] Li G, Ye T, Xia Z, Wang S, Zhu Z (2023). Analysis and prediction of hematocrit in microvascular networks. International Journal of Engineering Science.

[35] Kishimoto S, Maruhashi T, Kajikawa M, Matsui S, Hashimoto H, Takaeko Y (2020). Hematocrit, hemoglobin and red blood cells are associated with vascular function and vascular structure in men. Scientific Reports.

[36] Jamadar SD, Ward PGD, Liang EX, Orchard ER, Chen Z, Egan GF (2021). Metabolic and Hemodynamic Resting-State Connectivity of the Human Brain: A High-Temporal Resolution Simultaneous BOLD-fMRI and FDG-fPET Multimodality Study. Cereb Cortex.

[37] Sabatino L, Lapi D, Del Seppia C (2024). Factors and Mechanisms of Thyroid Hormone Activity in the Brain: Possible Role in Recovery and Protection. Biomolecules.

[38] Sawicka-Gutaj N, Zawalna N, Gut P, Ruchała M (2022). Relationship between thyroid hormones and central nervous system metabolism in physiological and pathological conditions. Pharmacological Reports.

[39] Li Y, Luan S, Ruan C, Li W, Zhang X, Ran Z (2024). TSHR signaling promotes hippocampal dependent memory formation through modulating Wnt5a/β-catenin mediated neurogenesis. Biochemical and Biophysical Research Communications.

[40] Eslami-Amirabadi M, Sajjadi SA (2021). The relation between thyroid dysregulation and impaired cognition/behaviour: An integrative review. Journal of Neuroendocrinology.

[41] Kim B, Moon SW (2020). Association between Thyroid Hormones, Apolipoprotein E, and Cognitive Function among Cognitively-Normal Elderly Dwellers. Psychiatry Investig.

[42] Chen R, Huang L, Wang R, Fei J, Wang H, Wang J (2024). Advances in Non-Invasive Neuromodulation Techniques for Improving Cognitive Function: A Review. Brain Sciences.

[43] Begemann MJ, Brand BA, Ćurčić-Blake B, Aleman A, Sommer IE (2020). Efficacy of non-invasive brain stimulation on cognitive functioning in brain disorders: a meta-analysis. Psychological Medicine.

