# Supplementary material for: Large-Scale Brain Network Connectivity Mediates the Association Between Metabolic Risk Factors and Cognition: An fMRI Study of the Human Connectome Project
Source: Int J Endocrinol Metab. 2026 Aug 11;24(4):e168867. doi: 10.5812/ijem-168867 (PMC13589462; doi:10.5812/ijem-168867)
Supplement: Supplementary file 1 [file ijem-24-4-168867-s001.pdf]

Table S1. The significant associations between metabolic risk factors (independent) and cognitive test scores or brain network connectivities (dependent) using multiple linear regression models.

Cognitive test score /networks connectivity	BMI			Hematocrit			TSH		
	β	SE	95% CI	β	SE	95% CI	β	SE	95% CI
PicVocab test score	-0.0698*	0.0349	-0.1385 to -0.0011	0.1159**	0.0401	0.0371 to 0.1948	ns	ns	ns
Gf test score	ns	ns	ns	ns	ns	ns	0.0950*	0.0370	0.0223 to 0.1677
Primary visual-salience	-0.1217**	0.0384	-0.1973 to -0.0461	ns	ns	ns	ns	ns	ns
Primary visual-auditory	0.1338***	0.0391	0.0570 to 0.2106	-0.1164*	0.0452	-0.2051 to -0.0276	ns	ns	ns
Primary visual-sensorimotor	0.0780*	0.0393	0.0006 to 0.1553	ns	ns	ns	ns	ns	ns
Primary visual-dorsal attention	-0.0789*	0.0395	-0.1566 to -0.0013	ns	ns	ns	ns	ns	ns
DMN-lateral visual	-0.1034**	0.0389	-0.1798 to -0.0270	ns	ns	ns	ns	ns	ns
DMN-language	0.0763*	0.0380	0.0016 to 0.1512	ns	ns	ns	ns	ns	ns
DMN-sensorimotor	0.1222**	0.0392	0.0450 to 0.1994	ns	ns	ns	ns	ns	ns
DMN-executive attention	0.1074**	0.0390	0.0308 to 0.1841	ns	ns	ns	ns	ns	ns
Lateral visual-sensorimotor	-0.0810*	0.0394	-0.1585 to -0.0036	ns	ns	ns	ns	ns	ns
Lateral visual-cerebellar	-0.0818*	0.0386	-0.1578 to -0.0058	ns	ns	ns	ns	ns	ns
Lateral visual-auditory	0.1563***	0.0391	0.0794 to 0.2332	ns	ns	ns	ns	ns	ns
Sensorimotor-frontoparietal	0.1177**	0.0381	0.0427 to 0.1927	ns	ns	ns	ns	ns	ns
Frontoparietal-language	0.1298***	0.0391	0.0528 to 0.2067	ns	ns	ns	ns	ns	ns
Frontoparietal-cerebellar	-0.0843*	0.0389	-0.1608 to -0.0078	ns	ns	ns	ns	ns	ns
Frontoparietal-auditory	0.1244**	0.0393	0.0470 to 0.2017	ns	ns	ns	0.0861*	0.0387	0.0101 to 0.1621
Salience-sensorimotor	0.1010*	0.0395	0.0234 to 0.1786	0.1264**	0.0454	0.0371 to 0.2157	ns	ns	ns
Salience-dorsal attention	0.1447***	0.0389	0.0682 to 0.2212	ns	ns	ns	ns	ns	ns
Language-ventral attention	0.1231**	0.0387	0.0470 to 0.1991	ns	ns	ns	ns	ns	ns
Language-executive attention	0.1199**	0.0390	0.0433 to 0.1965	ns	ns	ns	ns	ns	ns
Cerebellar-dorsal attention	-0.0860*	0.0390	-0.1627 to -0.0092	ns	ns	ns	ns	ns	ns
Auditory-ventral attention	0.1564***	0.0374	0.0828 to 0.2300	ns	ns	ns	ns	ns	ns
Medial visual-dorsal attention	0.0782*	0.0394	0.0008 to 0.1557	ns	ns	ns	ns	ns	ns
Primary visual-ventral attention	ns	ns	ns	-0.0999*	0.0452	-0.1887 to -0.0111	ns	ns	ns
DMN-auditory	ns	ns	ns	-0.1067*	0.0451	-0.1953 to -0.0181	ns	ns	ns
Frontoparietal-sensorimotor	ns	ns	ns	-0.0886*	0.0450	-0.1771 to -0.0002	ns	ns	ns
Salience-auditory	ns	ns	ns	-0.1197**	0.0452	-0.2086 to -0.0308	ns	ns	ns
Language-dorsal attention	ns	ns	ns	0.0967*	0.0451	0.0082 to 0.1853	ns	ns	ns

**Medial visual-ventral attention**	ns	ns	ns	0.0881*	0.0448	3.922e-005 to 0.1762	ns	ns	ns
**Primary visual-DMN**	ns	ns	ns	ns	ns	ns	0.0826*	0.0385	0.0070 to 0.1582
**Lateral visual-frontoparietal**	ns	ns	ns	ns	ns	ns	-0.0969*	0.0383	-0.1722 to -0.0216

Note: Standardized regression coefficients (β) are reported. Models are adjusted for age, sex, race, and education. "ns" = non-significant. *, **, and *** indicate p-values <0.05, <0.01, and <0.001, respectively.

Abbreviations: BMI, body mass index; TSH, thyroid-stimulating hormone; SE, standard error; CI, confidence interval; PicVocab, picture vocabulary; Gf, fluid intelligence; DMN, default mode network.
